# Evaluation of visual functions in Iranian hypothyroid adults

**DOI:** 10.1002/edm2.393

**Published:** 2022-12-14

**Authors:** Somayyeh Boomi Quchan Atigh, Habibe Sadat Shakeri, Habibollah Esmaily, Azam Darvishi, Aghdas Hamidi, Javad Heravian Shandiz

**Affiliations:** ^1^ Refractive Errors Research Center Mashhad University of Medical Sciences Mashhad Iran; ^2^ Department of Optometry, School of Paramedical Science Mashhad University of Medical Sciences Mashhad Iran; ^3^ School of Medicine North Khorasan University of Medical Sciences Bojnurd Iran; ^4^ Social Determinants of Health Research Center Mashhad University of Medical Sciences Mashhad Iran

**Keywords:** colour vision, contrast sensitivity, hypothyroidism, refractive errors

## Abstract

**Introduction:**

The aim of this study was to investigate the effects of hypothyroidism on visual functions such as visual acuity, refractive errors, colour vision, and contrast sensitivity, among hypothyroid adults.

**Methods:**

Forty‐three patients with clinical hypothyroidism along with 43 age‐ and sex‐matched healthy individuals underwent visual examinations, including visual acuity, refractive errors, eye deviations with the cover test, colour vision with the D15 test, and contrast sensitivity with Pelli‐Robson test.

**Results:**

It was indicated that visual acuity, refractive errors, phoria, and colour vision had no significant difference between the hypothyroid and control groups. Contrast sensitivity decreased in hypothyroid subjects as compared with controls. The mean values of binocular contrast sensitivity were 1.85 ± 0.09 log in the hypothyroid group and 1.93 ± 0.09 log in controls, which showed a statistically significant difference (*p* = .03).

**Conclusions:**

Our findings illustrated a reduced contrast sensitivity in adult hypothyroidism. Since CS is related to functioning and quality of life, a comprehensive and detailed eye examination may be beneficial for hypothyroidism patients.

## Graphical abstract

The reduction of stereopsis and contrast sensitivity in hypothyroid patients is indicative of defects in the magnocellular system. Hypothyroidism in adults can impair the visual function and influence the quality of life.

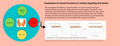

## 1 INTRODUCTION

Hypothyroidism is a common clinical disease caused by a decrease in thyroid hormone secretion from thyroid gland.^1^ This hormone has been known to regulate visual functions.^2^ Studies suggest that there is a probable relationship between hypothyroidism and age‐related macular degeneration.^3^, ^4^ Thyroid hormones play a critical role in neuroretinal maturation of the eye and development of the central nervous system (CNS). One of the thyroid hormone receptors on the retina is the β 2 receptor, which not only acts as an important regulator of opsin expression during cone photoreceptor development,^5^ but is also required for differentiation of cones versus rods, differentiation of cone subtypes, and production of essential proteins.^6^ The spectral identity and the retinal distribution of S‐ (short‐wave sensitive) and M‐ (middle‐ to long‐wave sensitive) cones are under rigid preservation to support visual functions, including contrast as well as colour vision.^7^ Abou‐Elghait et al. (2011) reported that thyroid hormone deficiency led to the reduced thickness of the retinal layer. Also, some structural changes were observed in the hypothyroid retinal cells, especially photoreceptors, inner nuclear layer, and ganglionic cell layer.^5^ Most studies have been conducted on visual function in infants with hypothyroidism.^6^, ^8^, ^9^, ^10^, ^11^, ^12^ However, there is some evidence that hypothyroidism can carry effects on the results of visual evoked potential (VEP) testing in adults.^13^, ^14^, ^15^ Moreover, it has been found that cone opsin expression is controlled by thyroid hormone throughout life, and alterations in serum levels of thyroid hormone may influence the adult retina and CNS, resulting in impairment of visual function.^7^, ^13^, ^14^, ^15^, ^16^ In this study, the status of visual function was assessed in adult individuals with hypothyroidism to answer the question whether the disease can affect visual performance such as visual acuity, contrast sensitivity and colour vision; it also provides insight into the diagnosis of the disease, further applications in future research, and allocation of health resources for the prevention of the disease complications in future.

## 2 METHODS

This case–control study was carried out on 43 hypothyroid patients aged 20–60 years who were admitted to Imam Reza Hospital in Bojnurd, Iran, as opposed to 43 age‐ and gender‐matched healthy controls. The research was approved at the Regional Ethics Committee of Mashhad University of Medical Sciences (Ethics Code: IR.MUMS.REC.1396.134). Also, written informed consent was taken from each participant. Based on the inclusion criteria, the case group composed of patients who were recently diagnosed as having clinical hypothyroidism, defined by higher TSH levels than the upper limit of normal and lower T4 levels than the normal range, and received no medication for the treatment. The presence of metabolic and systemic diseases that could affect the retina, contrast sensitivity, and colour vision, probability of any drugs with an influence on the visual functions, any ocular diseases, such as glaucoma, amblyopia, cataracts, dry eye, and retinal or macular degeneration, and history of ocular surgery ruled out the participation.

Initially, a thorough history was taken from the subjects, and then visual examinations including visual acuity, refractive errors measurement and its correction, fundoscopy, colour vision, and contrast sensitivity were performed. The best visual acuity with and without correction was monocularly measured using the standard Snellen chart at 6 m within a controlled illumination condition (100 cd/m^2^). Fundoscopy was conducted by applying Heine ophthalmoscope, and those participants who had posterior segment abnormalities were excluded from the study. Refractive errors were determined and subsequently corrected by automatic refractometr (Nidek AR‐610 autorefractometer), retinoscopy, and subjective method. Eye deviations were evaluated by the cover test and prism bar (20/30 “E” Snellen). The colour vision was measured by the ishihara and Lanthony Desaturated Panel D‐15) Clement Clarke International LTD Harlow, Essex, the United Kingdom) at 50 cm and in photopic illumination. The above‐mentioned tests were performed after the correction of refractive errors. Contrast sensitivity was explored employing a Pelli‐Robson chart (Clement ClarkInternational LTD Harlow, Essex, United Kingdom) under photopic and stable (120 cd/m^2^) illumination. Following correction, this test was run again for right, left, and both eyes, respectively. All these experiments were carried out for both case and control groups by the same investigator in the same environment and under equal conditions. In this study, levels of refractive errors were measured as spherical equivalent (sphere power + half negative cylinder power). Hyperopic refractive errors were considered as spherical equivalent of +0.5 diopters or more, and myopia was defined as spherical equivalent of −0.5 diopters or less.^17^ Astigmatism of 0.75 diopters or more was recorded with a minus sign.^18^ The astigmatic axis was classified as with‐the‐rule (between 0° and 30° or 150° or 180°), against‐the‐rule (between 70° and 130°), and oblique (between 0° and 30° or 150° or 180°).^19^

### 2.1 Statistical analysis

The data were collected and fed into SPSS software version 19 (IBM, USA). The difference between hypothyroid and healthy groups was determined using Fisher's exact test, chi‐square test, analysis of variance (ANOVA), and Kruskal–Wallis test. Numerical data were compared by independent samples *t*‐test, or its non‐parametric equivalent, and Mann–Whitney *U*‐test. A *p*‐value less than .05 was considered statistically significant.

## 3 RESULTS

This study included 86 participants (43 patients with hypothyroidism and 43 healthy controls). Table 1 summarizes the data concerning the comparison of age, sex, and TSH and T4 levels between the two groups. This study included 43 hypothyroid participants, 88.4% (*n* = 38) of whom were female and 11.6% of whom (*n* = 5) were male. The mean ages were 33.5 ± 9.5 years (range: 20–60 years) and 32.9 ± 8.5 years (range: 20–60 years) for the hypothyroid subjects and healthy controls, respectively. Both groups were not comparable in terms of age (*p* = .75, *t* = 0.324) and sex (*p* = .00, Exact fisher = 0.00).

**TABLE 1:** Demographic characteristics and levels of thyroid‐releasing hormone for both hypothyroid and control participants

	Hypothyroidism	Control	*p*
Age			
(mean + −SD)	33.5 ± 9.5	32.9 ± 8.5	.75
(min‐max)	20–59	21–58	
Gender			
Female	38(88.4%)	38(88.4%)	1.00
5(11.6%)	5(11.6%)		
T4			
(mean + − SD)	2.4 ± 1.4	7.6 ± 1.5	<.001
(min‐max)	0.3–4.5	4.9–10.1	
TSH			
(mean + − SD)	41.36 ± 66.3	2.8 ± 0.1	<.001
(min‐max)	8.2–367.5	1.1–4.8	

Abbreviations: TSH, thyroid‐stimulating hormone; T4, free thyroxine.

In the hypothyroid group, the mean values of uncorrected visual acuity were 0.82 ± 0.28 and 0.83 ± 0.28 for the right and left eyes, respectively. The healthy controls yielded values of 0.87 ± 0.23 and 0.87 ± 0.22 in the right and left eyes (the levels of visual acuity were in decimal form). Using Mann–Whitney *U* test, no significant differences were found between these values for the right (*p* = .636, *z* = −0.47) and left (*p* = .53, *z* = −0.62) eyes. For both groups, (corrected) visual acuity was fully corrected to 1.

In the hypothyroid group, the mean values of spherical equivalent were − 0.36 ± 1.4 D and − 0.34 ± 1.4 D for the right and left eyes, respectively. The healthy controls showed values of 0.1 ± 0.9 D and 0.2 ± 0.9 in the right and left eyes. Despite the high prevalence of hyperopia in the healthy controls and myopia in the hypothyroid subjects, there was no statistically significant difference between refractive errors in the two groups based on chi‐square analysis (*χ* ^2^ = 0.489, *p* = .78). Mean levels of astigmatism were observed 0.66 ± 0.7 D in the hypothyroid group and 0.66 ± 0.5 D in the control group. The findings of Mann–Whitney U test indicated that no considerable difference occurred between the two (*z* = 0.39, *p* = .69 for the right eye and *z* = 0.71, *p* = .47 for the left eye). Most of the patients with hypothyroidism and the healthy subjects were with‐the‐rule astigmatism. Of the case group, 51.3% (*n* = 20), 25.6% (*n* = 10), and 23.1% (*n* = 9) developed with‐the‐rule, against‐the‐rule, and oblique astigmatism, respectively. On the basis of chi‐square analysis, the hypothyroidism group was not notably different (*χ* ^2^ = 0.97, *p* = .62) from the control group, where with‐the‐rule, against‐the‐rule, and oblique astigmatism was, respectively, reported in 17 (41.5%), 11 (26.8%), and 13 (31.7%) participant.

As shown in Table 2, according to the results of Fisher's exact test, there was no significant difference in far (Fisher's exact = 0.345, *p* = .00) and near (Fisher's exact = 1.07, *p* = .74) types of heterophoria for both groups.

**TABLE 2:** Visual functions of the hypothyroid and control groups

		Hypothyroidism	Control	*p*
Contrast sensitivity(right eye)				
(mean ± SD)		1.69 ± 0.1	1.71 ± 0.09	.045
(min‐max)		1.45–1.9	1.45–1.9	
Contrast sensitivity (left eye)				
(mean ± SD)		1.66 ± 0.1	1.70 ± 0.09	.03
(min‐max)		1.4–1.9	1.4–1.9	
Contrast sensitivity (binocular)				
(mean ± SD)		1.85 ± 0.09	1.93 ± 0.09	<.001
(min‐max)		1.65–2.00	1.65–2.15	
Emmetropia *N* (%)		16 (37.2)	15 (34.9)	.05<
Myopia *N* (%)		13 (30.2)	11 (25.6)	
Hyperopia *N* (%)		14 (32.6)	17 (39.5)	
Far	Orthophoria *N* (%)	41 (95.3)	42 (97.7)	1/00
	Exophoria *N* (%)	2 (4.7)	1 (2.3)	
	Esophoria *N* (%)	0 (0.0)	0 (0.0)	
Near	Orthophoria *N* (%)	17 (39.5)	13 (30.2)	0/74
	Exophoria *N* (%)	25 (58.1)	29 (67.4)	
	Esophoria *N* (%)	1 (2.3)	1 (2.3)	
Colour vision	Normal *N* (%)	37 (86.0)	(95.3) 41	0.2
	Mild Tritan *N* (%)	(7.0) 3	(0.0) 0	
	Mild Deutan *N* (%)	(7.0) 3	(4.7) 2	

The mean values of the near point of convergence (a 20/30 letter as the target on a near chart) were 8.7 ± 2.1 cm for the hypothyroid cases and 8.7 ± 3.1 cm for the peer controls which had no significant difference between the two groups (*p* = .98, *t* = 0.02).

All participants were tested by the ishihara and appeared to have normal colour vision; however, the D15 detected some cases with mild defects. The results of Fisher's exact test demonstrated that there was no significant difference in colour vision defects (Fisher's exact = 3.07, *p* = .2) between the study groups. The mean levels of TSH were 18.99 ± 47.46 mIU/L in normal colour vision individuals, 17.04 ± 14.17 mIU/L in subjects with mild deutan defect, and 37.28 ± 68.2 mIU/L in those with mild tritan defect, which were not considerably different from one another (*χ* ^2^ = 2.89, *p* = .08) by using the Kruskal–Wallis test. The participants with normal colour vision, mild deutan defect, and mild tritan defect, respectively, displayed mean T4 levels of 5.58 ± 3.35, 4.69 ± 2.99, and 2.81 ± 1.91 μg/dL, indicating no marked difference among them based on ANOVA analysis (*p* = .32, *F* = 1.14).

The monocular and binocular contrast sensitivities of the hypothyroid group were significantly lower than the control group. Applying the receiver operating characteristic (ROC) curve, the sensitivity and specificity of contrast sensitivity were calculated. It was found that contrast sensitivity was effective factor for hypothyroidism (*p* < .05). The critical value was <1.6 log for monocular contrast sensitivity and <1.9 log for binocular contrast sensitivity.

## 4 DISCUSSION

This study intended to investigate the status of visual function in adult hypothyroid patients. Despite the large number of studies regarding hypothyroidism in children and infants, few studies have investigated the visual functions in adult patients.

In this study, all participants had similar corrected visual acuity (20/20), and corrected and uncorrected VA was not comparable in two groups. There was no significant difference in refractive errors (myopia, hyperopia, and astigmatism) between the two groups. Although there has been no study with the same age range and method as ours, the existing literatures on participants with different age categories and or using VEP for evaluating visual functions have exhibited that hypothyroidism appears not to affect refractive errors, visual acuity, manifest and latent strabismus, and NPC.^6^, ^11^, ^20^, ^21^

The results showed the difference in contrast sensitivity in two groups; thyroid dysfunction is associated with neurological and behavioural abnormalities in adults.^22^ Variations in serum thyroid hormone are more likely to influence the adult human retina, of note cone opsin expression, and impair visual function, particularly the function of magnocellular and parvocellular cells related to contrast sensitivity.^7^ Indeed, this is a psychophysical study indicating that contrast sensitivity in hypothyroid cases is lower than that in peer controls. Similarly, from an electrophysiological perspective, several studies have been conducted concerning hypothyroidism in adults and presented that the VEP latency increased and yet the P100 amplitude decreased in these patients.^13^, ^14^, ^15^, ^23^) Aprachita and Aggarwal (2017) have attributed these two events to defects in myelination and impaired axonal function, respectively.^23^) Considering the role of ganglion cells in contrast sensitivity,^24^, ^25^) Holder and Condon (1989) demonstrated that pattern electroretinogram was subnormal in adult hypothyroid patients, probably due to the ganglion cell dysfunction.^26^) Thyroid hormone carries effects on the CNS via gene expression, myelin synthesis, axonal transportation, and neurotransmission.^23^) Given that the normal function of contrast sensitivity depends on the integrity of neurons and visual cortex,^27^, ^28^) any disturbance in the regions and neuronal function can cause impacts on contrast sensitivity. In adults with hypothyroidism, alterations in synaptic transmission and its plasticity, and loss of neurons in the hippocampus, as well as fluctuations in interneurons have been recorded.^29^) In the light of these findings that a lack of thyroid hormone has the ability to impair the mature retina and mature nerve cells, and visual structures from the retina to the cortex are involved in contrast sensitivity,^27^, ^28^, ^30^ decline of contrast sensitivity found in the present study may arise from defects in photoreceptors or retinal ganglion cells or magnocellular neurons in the lateral geniculate nucleus or besides decreased production of myelin. However, further studies are needed to explain more the nature of CS function and its effects on vision in adults with hypothyroidism. The more reduction in binocular contrast sensitivity compared with monocular one in the hypothyroid patients versus their peer controls may imply that the mechanisms and region of visual cortex binocular neurons are affected to a greater extent by hypothyroidism, which warrants further investigations. More to the point, since binocular test results denote the individual's quality of daily life, decline of binocular contrast sensitivity in hypothyroid patients can compromise the quality of their lives.

In this study, 7% of the hypothyroid individuals possessed mild deutan defects and another 7% presented with mild tritan defects. However, no statistically significant difference was observed between the two groups in this regard. The studies have reported conflicting evidence with respect to the role of thyroid hormone in the mature cone photoreceptors. Umali et al. (2014) conducted a study on the relationship between hypothyroidism and colour vision defects in Filipino adults using the ishihara and D15. They showed that of 37 hypothyroid patients, only one possessed deutan defects that might be due to genetic deficiency, not hypothyroidism. Indeed, they concluded that hypothyroidism carries no effect on colour vision because cones of the adult human are not easily affected by changes in levels of thyroid hormones.^31^ In consistent with these findings by Umali et al. (2014), there was no marked disparity in colour vision between the two groups in our study. However, we could detect mild defects in colour vision that might be related to the use of the LDT D15 test, where colours are less saturated thus even mild defects in colour vision can be better distinguished. By contrast, Cakir et al. (2015) employed Chromatest to examine colour vision in adults with hypothyroidism. They reported both defects in yellow‐blue and red‐green vision, with the former being more impaired among them. Furthermore, a significant difference was found between hypothyroidism and colour contrast sensitivity.^32^ The cause underlying the difference between these results and ours may be the type of test used, implying that Chromatest has higher sensitivity for identifying colour vision defects.

Animal studies suggest that thyroid hormone represses the expression of S opsin through β_2_ receptor and yet stimulate the expression of M opsin.^7^, ^33^, ^34^ Therefore, we expected that protan defects become greater than tritan ones among hypothyroid patients, which is in opposition to Cakir et al. (2015)'s findings and ours.^32^ In mice, M and S opsins have been found to be largely distributed in the dorsal and ventral retina, respectively. On the contrary, cones follow a mosaic‐like pattern in rats so that S cones contain a minority of the population (10% of the total retina).^7^ When it comes to human, the foveola comprises only L and M cones, while S cones with a mosaic‐like pattern are present outside the foveola within the fovea and the macula. Rods are prevailing in the posterior pole in which S and M cones scatter throughout with a random pattern.^35^ Accordingly, the only explanation for the difference in the type of colour vision defects between human and animal models may be due to the difference in opsin expression between retinae of trichromatic human and dichromatic mouse. Although the D15 test is common in clinical examinations, there is a lack of gold standard for colour vision assessment. Distinguishing between S and M responses is so difficult, but it is likely that the use of electroretingram with colour stimuli can elicit more information in this regard.

This study has some limitations as follow: the duration of hypothyroidism is unknown and we failed to obtain relevant data; the sample size was small because all the patients did not have T4 findings.

Our study presented that hypothyroidism might diminish contrast sensitivity, thereby affecting the quality of life. Decline of contrast sensitivity seen in hypothyroid patients compared with controls may effectively contribute to the diagnosis, control, and prevention of the disease.

## 5 CONCLUSIONS

In this study, contrast sensitivity decreased in the patients with hypothyroidism during adulthood. Hence, contrast sensitivity test can be regarded as highly sensitive diagnostic tool for evaluating visual functions among hypothyroid subjects, which, however, require further studies on a large sample size.

## AUTHOR CONTRIBUTIONS

**Somayyeh Boomi Quchan Atigh:** Investigation (lead); project administration (equal); visualization (equal); writing – original draft (lead). **Habibe Sadat Shakeri:** Conceptualization (equal); investigation (equal); validation (supporting); visualization (supporting). **Habibollah Esmaily:** Formal analysis (lead); methodology (lead); software (supporting). **Azam Darvishi:** Methodology (equal); project administration (supporting); resources (equal); writing – review and editing (supporting). **Aghdas Hamidi:** Data curation (supporting); investigation (supporting); resources (supporting); validation (supporting).

## CONFLICT OF INTEREST

The authors have no conflict of interest with the subject matter of the present study.

## Data Availability

Data sharing is not applicable to this article as no new data were created or analyzed in this study.
